# Natural killer-like B cells are a distinct but infrequent innate immune cell subset modulated by SIV infection of rhesus macaques

**DOI:** 10.1371/journal.ppat.1012223

**Published:** 2024-05-13

**Authors:** Cordelia Manickam, Amit A. Upadhyay, Griffin Woolley, Kyle W. Kroll, Karen Terry, Courtney A. Broedlow, Nichole R. Klatt, Steven E. Bosinger, R. Keith Reeves

**Affiliations:** 1 Division of Innate and Comparative Immunology, Center for Human Systems Immunology, Duke University, Durham, North Carolina, United States of America; 2 Department of Surgery, Duke University, Durham, North Carolina, United States of America; 3 Department of Pathology and Laboratory Medicine, Emory University School of Medicine, Atlanta, Georgia, United States of America; 4 Division of Microbiology and Immunology, Emory National Primate Research Center, Atlanta, Georgia, United States of America; 5 Division of Surgical Outcomes and Precision Medicine Research, Department of Surgery, University of Minnesota, Minneapolis, Minnesota, United States of America; NIH, NIAID, UNITED STATES

## Abstract

Natural killer-like B (NKB) cells are unique innate immune cells expressing both natural killer (NK) and B cell receptors. As first responders to infection, they secrete IL-18 to induce a critical cascade of innate and adaptive immune cell infiltration and activation. However, limited research exists on the role of NKB cells in homeostasis and infection, largely due to incomplete and erroneous evaluations. To fill this knowledge gap, we investigated the expression of signaling and trafficking proteins, and the *in situ* localization and transcriptome of naïve NKB cells compared to conventionally-defined NK and B cells, as well as modulations of these cells in SIV infection. Intracellular signaling proteins and trafficking markers were expressed differentially on naïve NKB cells, with high expression of CD62L and Syk, and low expression of CD69, α4β7, FcRg, Zap70, and CD3z, findings which were more similar to B cells than NK cells. CD20^+^NKG2a/c^+^ NKB cells were identified in spleen, mesenteric lymph nodes (MLN), colon, jejunum, and liver of naïve rhesus macaques (RM) via tissue imaging, with NKB cell counts concentrated in spleen and MLN. For the first time, single cell RNA sequencing (scRNAseq), including B cell receptor (BCR) sequencing, of sorted NKB cells confirmed that NKB cells are unique. Transcriptomic analysis of naïve splenic NKB cells by scRNAseq showed that NKB cells undergo somatic hypermutation and express Ig receptors, similar to B cells. While only 15% of sorted NKB cells showed transcript expression of both KLRC1 (NKG2A) and MS4A1 (CD20) genes, only 5% of cells expressed KLRC1, MS4A1, and IgH/IgL transcripts. We observed expanded NKB frequencies in RM gut and buccal mucosa as early as 14 and 35 days post-SIV infection, respectively. Further, mucosal and peripheral NKB cells were associated with colorectal cytokine milieu and oral microbiome changes, respectively. Our studies indicate that NKB cells gated on CD3^-^CD14^-^CD20^+^NKG2A/C^+^ cells were inclusive of transcriptomically conventional B and NK cells in addition to true NKB cells, confounding accurate phenotyping and frequency recordings that could only be resolved using genomic techniques. Although NKB cells were clearly elevated during SIV infection and associated with inflammatory changes during infection, further interrogation is necessary to acurately identify the true phenotype and significance of NKB cells in infection and inflammation.

## Author summary

Recently, our understanding of traditional innate immunity has become reshaped by the discovery of new innate immune cell subsets as well as newly identified functions/programming of previously described innate immune cell subsets. One such new and unique innate immune cell subset are the natural killer-like B (NKB) cells, which express both NK and B cell receptors. These cells have been described as first responder immune cells in infection and inflammation within mice, non-human primates (NHP), and humans. To clarify if the simple definition of CD3^-^NKG2A/C^+^CD20^+^ cells as NKB cells is sufficient to truly identify the unique NKB cell phenotype, we characterized these cells via imaging cytometry and single cell RNA sequencing for the first time in an NHP model. Our data suggests that the NKB cell phenotype and transcriptome, while unique and relevant in SIV infection, remains very infrequent. Therefore, using flow cytometry to identify NKB cells based on the commonly accepted marker expression could be misleading and cause the erroneous classification of conventional NK cells and/or B cells as NKB cells.

## Introduction

Mounting evidence underscores the significance of innate immunity in the early control of human immunodeficiency virus (HIV)-1 and simian immunodeficiency virus (SIV) infection, preceding adaptive immune responses. Natural killer-like B (NKB) cells represent one innate subset profoundly impacted by HIV/SIV infection [1–4], though uncertainties around their identity and antiviral functions persist. A traditional understanding of the innate immune response primarily focuses on the role of dendritic cells (DCs) as rapid responders, detecting viral products through pattern recognition receptors (PRRs) and prompting the release of inflammatory cytokines that establish an antiviral state and activate other innate immune cells [5]. Natural killer (NK) cells are recognized as crucial innate mediators of antiviral control which mediate cytotoxicity by engagement of CD16 and/or due to the activation of their killer immunoglobulin-like receptors (KIRs) when viral infected cells lack major histocompatibility complex-I [6]. During acute HIV-1 infection, infected cells’ pathogen-associated molecular patterns engage PRRs, initiating intracellular antiviral defenses that curb viral replication. This response propagates outward through secreted factors like cytokines and chemokines, recruiting innate immune cells to infection sites and local lymphatic tissues. Antiviral innate effector cells not only aid in viremia control, but also shape the adaptive immune response to HIV-1. The interplay of PRR signaling, viral-restriction factors, innate immune cells, innate-adaptive immune communication, and viral evasion strategies collectively determines the course of HIV-1 infection and immune responses [7]. Various innate immune cells, including monocytes/macrophages, dendritic cells, NK cells, NKT cells, γδ T cells, B1 cells, mast cells, and granulocytes, remain active from early HIV infection to advanced AIDS stages [8].

In recent years, innate immunity has been reshaped by the discovery of novel immune subsets and novel functions of previously described innate immune cells. One such novel innate immune cell subset is the so-called NKB cells that express the receptors of both NK cells and B cells and was initially described in mice in 2016[9], as well as in primates in subsequent years [1,2,4]. Our own group has previously identified NKB cells existing in both RM and humans, and demonstrated their dysregulation over the course of HIV/SIV infection [1]. While the full functional niche of NKB cells remains unknown, their unique phenotype and systemic distribution could make them unique targets for immunotherapeutics or vaccine strategies [1]. Outside of our group, NKB cells have been identified within the colon of SIV-infected RM and cynomolgus macaques as a lymphocyte subset which has the properties and functions of NK and B cells while remaining a distinct cell population [2,9]. Indeed, NKB cells have been identified as functionally relevant to the occurrence of inflammation within the colon of NHP via their increased proliferation and production of inflammatory cytokines such as interleukin-18 (IL-18) during SIV infection [2,3]. Furthermore, NKB cells have been shown to exist within human spleen and MLN, and have been observed to activate other innate lymphocytes via secretion of IL-18 and IL-12 to mediate the inflammatory response to microbial infection [4,9]. Bone marrow derived mesenchymal stem cells have been demonstrated to normalize the increased proportions of NKB cells in the spleen which occur as a result of alcohol-induced organ injury [10].

NKB cells have been demonstrated to function as a separate subset of “innate-like B cells” (ILB) [11]. Substantial evidence supports the existence of ILB as multiple B cell subsets that are phenotypically and functionally distinct from conventional B cells and which facilitate the innate immune response and serve as a bridge between the innate and adaptive components of the immune system [12–14]. ILB, particularly the B-1 and marginal zone (MZ) subsets, are the primary source of natural immunoglobulin (Ig)M production [15–18]. Among the similarities between NKB cells and B cells is the expression of receptors and ligands with roles in antigen presentation and recognition, as well as class switching, affinity maturation, and B cell memory formation in secondary lymphoid follicles [2]. NKB cells primarily express IgA, and to a lesser extent IgM and IgG [2]. NKB cells also express NK cell activation receptors, Fas ligand, perforin, and granzymes and are capable of lysing cells [2]. Additionally, NKB cells produce the inflammatory cytokines, interferon gamma (IFN-γ), tumor necrosis factor alpha (TNF-α), and IL-18[2]. Finally, NKB cells have an increased proliferation capacity in comparison to NK and CD8^+^ T cells within RM SIV-infected colon [2].

The current literature on NKB cells, while continually expanding, remains limited, leaving several aspects of NKB function, distribution, and lineage shrouded in uncertainty. Indeed, others have questioned the true phenotype of NKB cells, instead suggesting that many types of immune cells (NK1.1^+^CD19^+^, NKp46^+^CD19^+^, and MZ B cells) might be erroneously identified as NKB cells [19]. With a preliminary understanding of innate immunology established, we decided to utilize an RM model of HIV infection to study NKB cells in naïve and infected NHP. We evaluated NKB cells in mucosal tissues of the gut of RM and observed elevated NKB frequency in buccal and colonic tissues of SIV-infected animals. However, to truly grasp the significance and role of NKB cells in infection, it is imperative to comprehend their homeostatic distribution and relevance in systemic, lymphoid, and mucosal tissues. To bridge this knowledge gap regarding the unique biology of NKB cells, we conducted an analysis of NKB cells in comparison to their B and NK cell counterparts within the peripheral blood and tissues of naïve RM using multiparametric flow cytometry, imaging cytometry, and transcriptomics. Our data suggests that the genuine NKB cell phenotype is rare, even in the spleen, which is enriched in ILB [20], even though mucosal expansion of NKB cells were observed in the buccal and colon tissues of SIV-infected RM. Further, NKB cells in SIV-infected RM were associated with pro-inflammatory cytokines and microbiome changes in gut and buccal mucosa. However, accurate identification of NKB cells is confounded by trancriptomically conventional B and NK cells expressing NK and B cell receptors, respectively. Given the potential functions of NKB cells in the immunopathogenesis of viral infections, the current determination of NKB cell phenotype via flow cytometric markers must be re-evaluated to exploit these cells for therapeutic targeting.

## Results

### Peripheral NKB cells express signaling and trafficking markers similar to conventional B cells in homestasis

**Fig 1:**
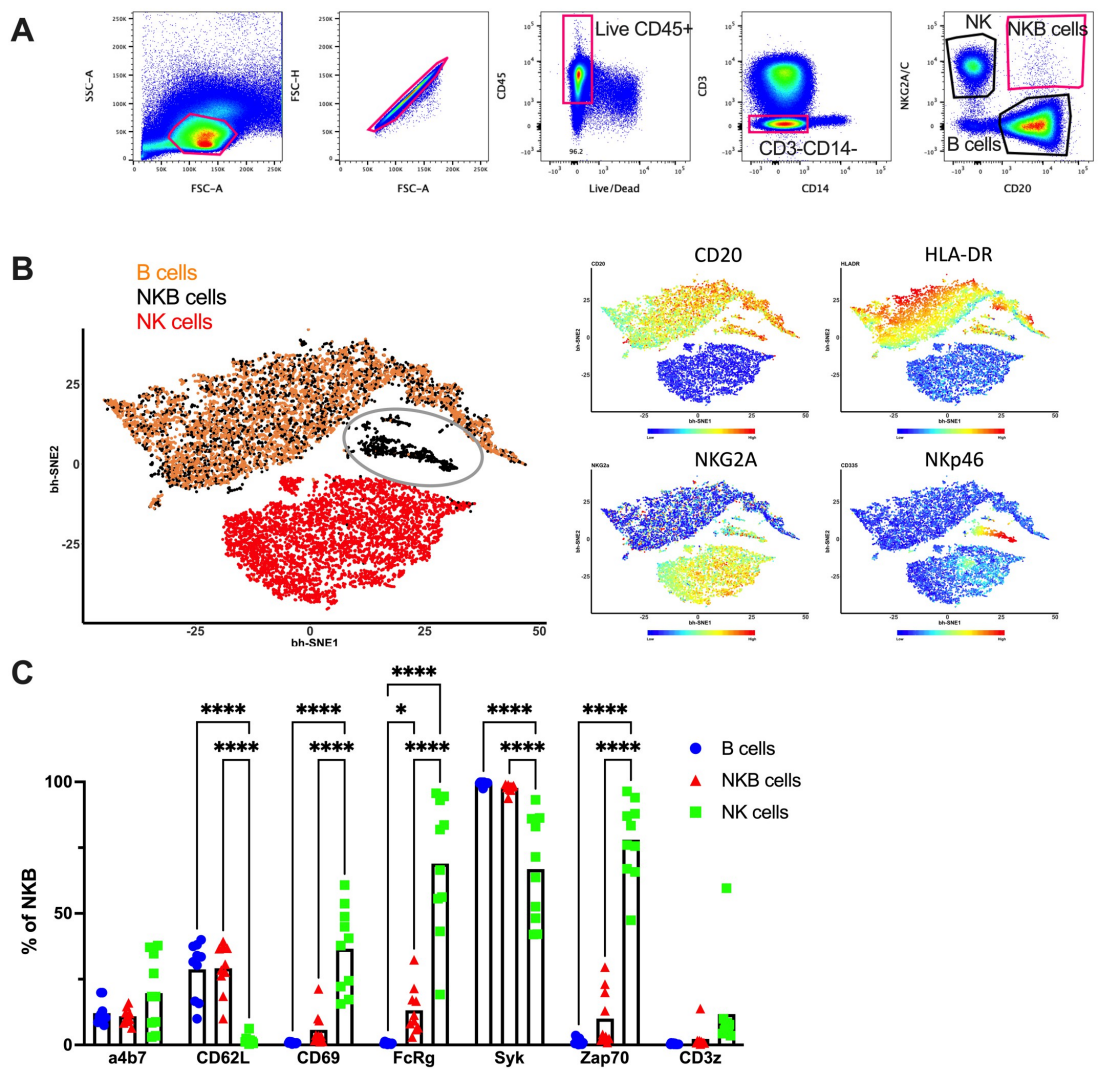
NKB cell phenotype in peripheral mononuclear blood cells (PBMC) of naïve rhesus macaques (RM). PBMC from naïve RM were stained for flow cytometry. **(A)** Gating strategy for identifying NKB, B, and NK cells by flow cytometric analaysis; **(B)** Multidimensional analysis of peripheral NKB, B, and NK cells via 30 parameter flow cytometric data analysis (**Left**) and marker overlay (**Right**); PBMC were stained and analyzed for **(C)** signaling and trafficking markers on NKB cells in comparison to B and NK cells (n = 10). Statistical differences between marker expression of NKB cells, B cells and NK cells were analyzed by two-way ANOVA test. * indicates *p*-value < 0.05, **** indicates *p*-value < 0.0001.

Using flow cytometry, we identified NK cells as CD3^-^CD14^-^CD20^-^NKG2A/C^+^ cells, B cells as CD3^-^CD14^-^CD20^+^NKG2A/C^-^ cells, and NKB cells as CD3^-^CD14^-^CD20^+^NKG2A/C^+^ cells from peripheral mononuclear blood cells (PBMC) of naïve RM (**Fig 1A and S1 Table**). Further data analysis with Barnes-Hut-Stochastic Neighbor Embedding (bh-SNE) showed the separation of NKB, B, and NK cell clusters (gray circle represents NKB cell cluster; **Fig 1B**), indicating a unique NKB cell phenotype. Immune cell function is mediated by phosphorylation of intracellular signaling adapters, which are differentially expressed in different immune cell subtypes. To understand the intracellular signaling machinery of NKB cells, we analysed the expression pattern of multiple signaling proteins in PBMC by flow cytometry. While peripheral NK cells expressed multiple activator signaling molecules, including FcRg, Syk, Zap70, and CD3z, peripheral B cells expressed only high levels of Syk. Interestingly, the peripheral NKB cells also showed high Syk and low FcRg and Zap70, which was similar to B cell signaling proteome expression (**Fig 1C**). Similarly, we analysed the trafficking protein profile of NKB cells in PBMC in comparison to NK and B cells. NKB cells expressed high CD62L and low CD69 proteins in a pattern similar to B cells and opposed to the NK cell trafficking phenotype. Statistical analysis also further confirmed that expression levels of all trafficking and signaling proteins studied on B cells and NKB cells, except a4b7 and CD3z, were significantly different from NK cells (**Fig 1C**). Collectively, these data indicate that the majority of NKB cells express trafficking markers more similar to conventional B cells than NK cells.

### Unique NKB cell phenotype identified in naive tissue compartments

**Fig 2:**
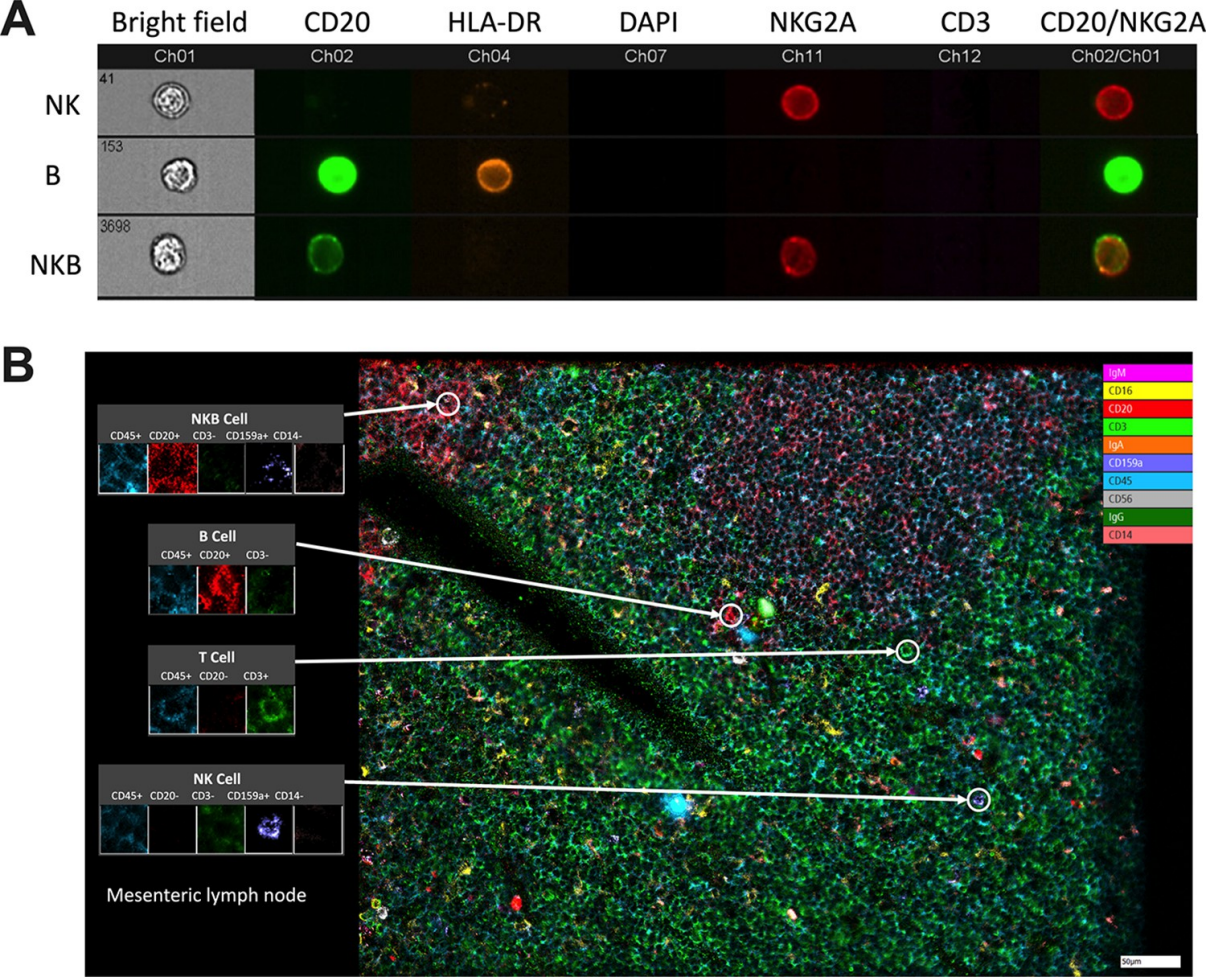
NKB cell phenotype in tissues of naïve rhesus macaques (RM). **(A)** Imagestream analysis of splenic mononuclear cells showing co-expression of CD20 and NKG2A/C. **(B)** *In-situ* localization of immune cells using ChipCytometry was done by subjecting tissue sections to multiple rounds of staining with antibodies, imaging, bleaching by Zellscanner microscope and data analyzed using ChipCytometry and ImageJ. NKB cells were visualized in mesenteric lymph node tissue. CD3, bright green; CD14, pink; CD16, yellow; CD20, red; CD45, light blue; CD56, gray; CD159a, dark blue; IgA, orange; IgG, dark green; IgM, magenta) of naïve RM.

**Fig 3:**
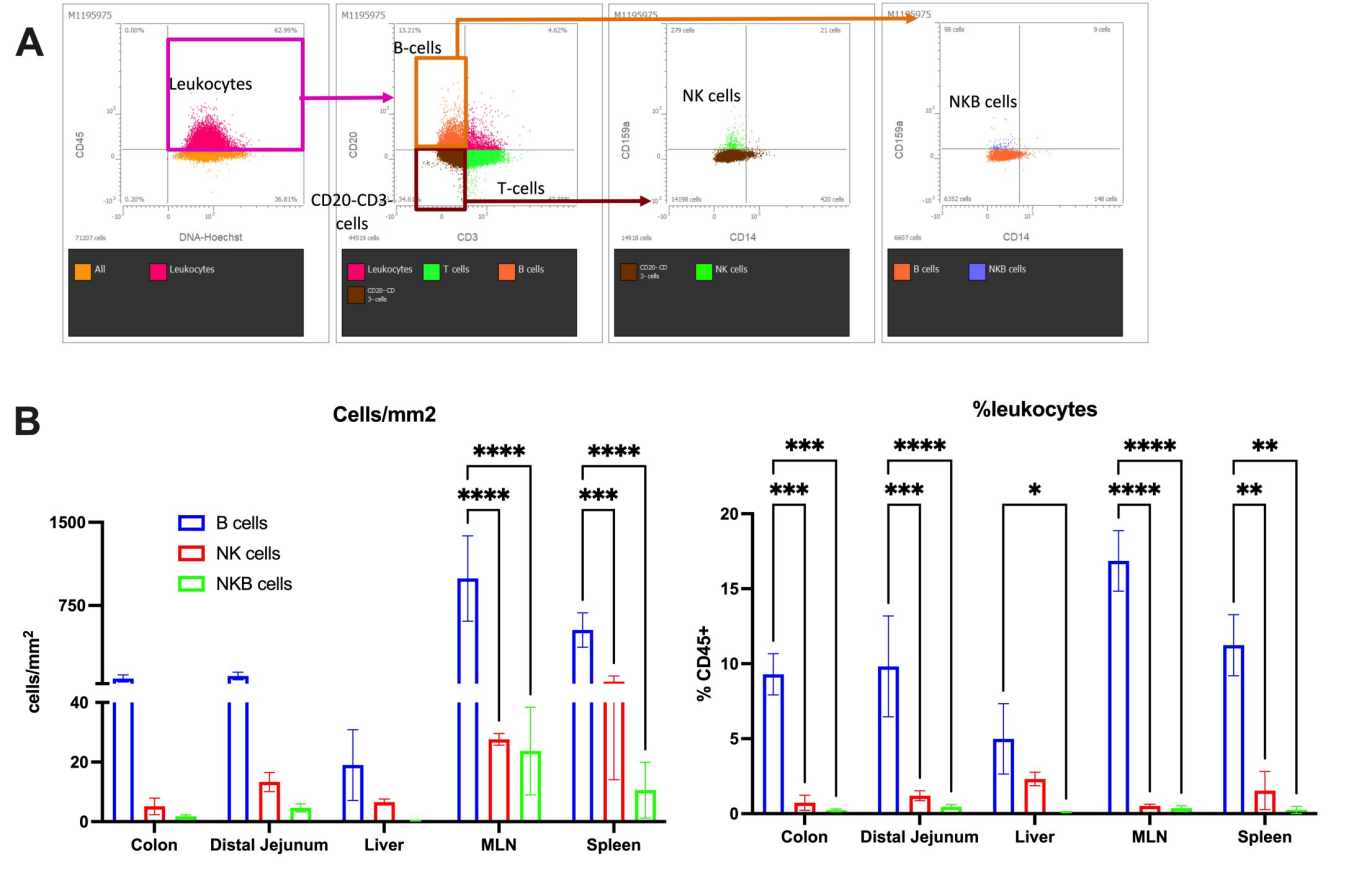
Imaging data analysis of NKB cells in tissues of naive rhesus macaques (RM). Imaged data from tissues of naïve RM were analysed using ZKW software of ChipCytometry. **(A)** Representative gating strategy used for identifying NK, B, and NKB cells in tissues and flow-based data analysis of stained tissue samples measuring **(B)** cells/mm^2^ and percentage of leukocytes (%CD45^+^), within colon, distal jejunum, liver, mesenteric lymph nodes (MLN), and spleen tissues of naïve RM (n = 2–4). Statistical differences between cells/mm^2^ and percentages of NKB, B, and NK cells were analyzed by multiple Mann-Whitney test. * indicates q-value < 0.05, ** q-value <0.01, *** q-value <0.001, **** q-value <0.0001.

Many studies have observed NKB cells to be enriched within various tissues, including the spleen and mucosae [1,3,9]. To rule out the potential nonspecific binding of antibodies, mononuclear cells from RM spleen were stained and analyzed by ImageStream cytometry. Single mononuclear cells expressing both NKG2A and CD20 were visualized (**Fig 2A**), indicating the presence of true NKB cells in spleen. Using 10-plex antibody imaging (**S2 Table**) via ChipCytometry on the frozen tissues of naïve RM, we visualized *in situ* NKB cells in MLN (**Fig 2B**) based on their co-expression of CD20 and NKG2A/C. Individual imaging analysis of naïve MLN tissues showing expression of immune cell markers and DNA dye are shown in (**S1 Fig**). Similar imaging of colon, liver, and jejunum sections from naïve RM showed the presence of NKB cells in these tissues (**Fig 3B**). The imaging data from multiple tissues was utilized to analyze single cell cytometric data to confirm the distribution of NKB, B and NK cells. Using ChipCytometry, dotplots were generated and immune cells were gated for NKB, B, and NK cells based on the marker expression of imaged tissue as in (**Fig 3A**). Immune cell counts and frequencies from the imaged data showed higher average NKB cell counts in MLN and spleen at 23.71 and 10.55 cell count/mm^2^ tissue area, respectively, in comparison to other tissues (**Fig 3B**). While NKB cells were significantly fewer than B cells in naïve colon, jejunum, and liver (**Fig 3B**), the sum of our imaging data corroborates previous findings of NKB cell enrichment in spleen and MLN as described in mice by Wang *et al*.[9].

### Single-cell transcriptomes of splenic NKB cells rarely contain both B and NK cell transcripts

**Fig 4:**
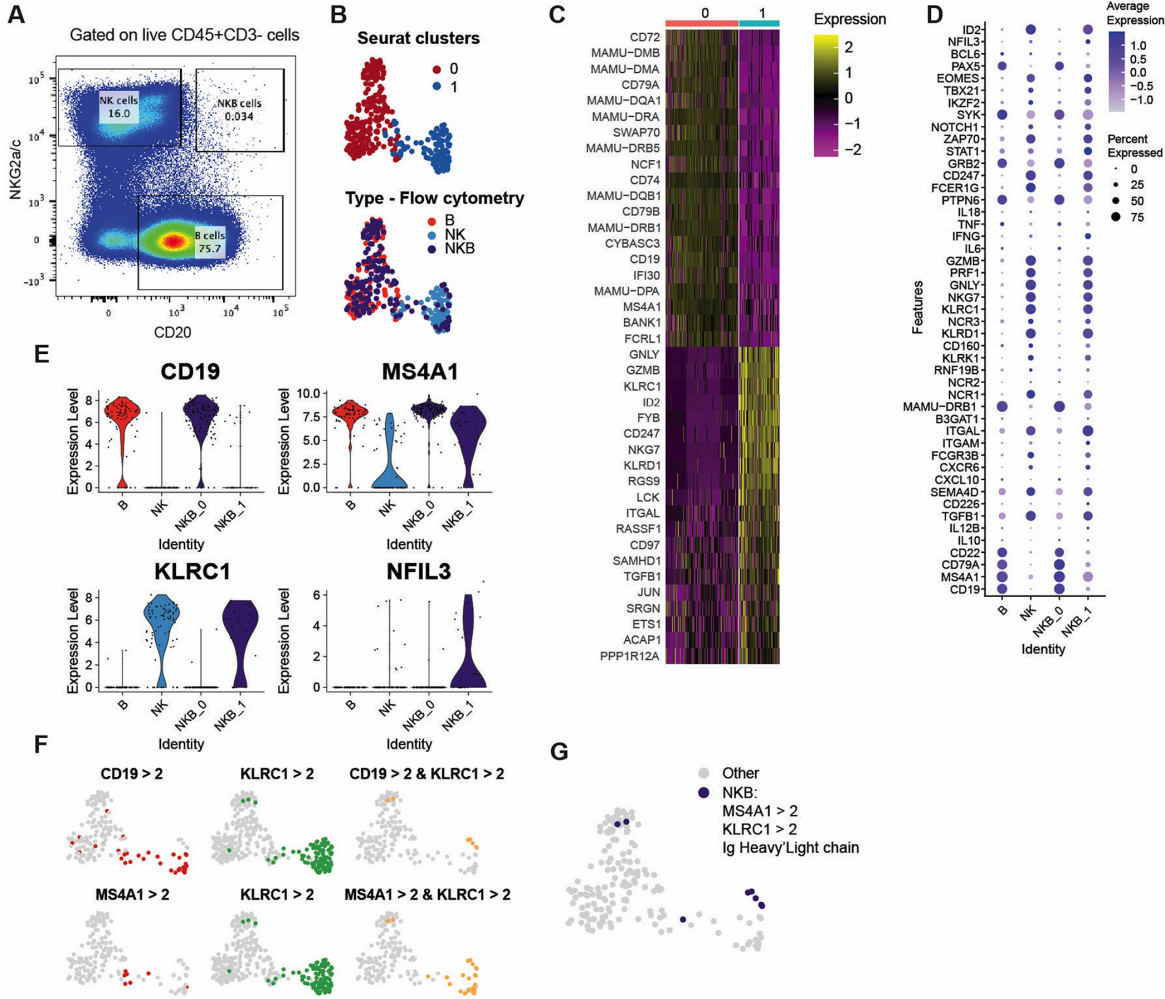
scRNA-Seq analysis of sorted NKB, B, and NK cells. **(A)** Representative flow plot showing sorting of NKB, B, and NK cells. **(B)** UMAP of 314 single cells (NKB cells– 159, B cells– 78, NK cells—77) from three rhesus macaques colored by seurat cluster and cell type. **(C)** Heat map showing the top twenty differentially-expressed genes ranked by average log2 fold-change between seurat cluster 0 and 1 (padj < 0.05 and expressed in 80% of cells from either group). **(D)** Dot plots showing expression of canonical B and NK cell marker genes in each of the cell types. The NKB cells from cluster 0 and 1 are shown separately. **(E)** Violin plots showing expression of NKB cell marker genes in each of the cell types. The NKB cells in cluster 0 and 1 are shown separately. **(F)** Co-expression of CD19, CD20/MS4A1, and NKG2A/KLRC1 in NKB cells. Cells expressing genes above the threshold are colored in red/green/yellow. **(G)** UMAP of NKB cells colored by cells that were found to express NKB marker genes and also express productive Ig heavy/and or light chain.

The majority of studies characterizing NKB cells have employed flow cytometry and bulk RNA-sequencing (RNA-seq). However, no study to date has employed single-cell RNA-Seq (sc-RNA-Seq) to accomplish this. To understand the transcriptional nature of NKB cells at single-cell resolution, we sorted CD45^+^CD3^-^NKG2A/C^+^CD20^+^ NKB cells, CD45^+^CD3^-^NKG2A/C^-^CD20^+^ B cells (total B lymphocytes which were comprised of both memory and naïve B cells), and CD45^+^CD3^-^NKG2A/C^+^ NK cells (**Figs 4 and** **S2**) and subjected the lysates to Smart-Seq-based library preparation (GEO accession: GSE263399). The rationale for choosing this method over more commonly used droplet-based methods (e.g. 10X Genomics) was three-fold: (i) the vastly improved signal in Smart-Seq allows for a high level of confidence to determine if a transcript is expressed or absent on a ‘per cell’ basis, and this resolution is not possible with droplet-based technology due to its inherently high drop-out level [3]; (ii) the rarity of NKB cells necessitates a high efficiency recovery method; and (iii) this approach allows for sequencing across the full-length of the transcript.

When clustered using Seurat analysis, the sc-RNA-Seq data showed two easily distinguishable clusters, which were denoted as Cluster 0 and Cluster 1 (**Fig 4B**, top panel). Upon examining the markers distinguishing these clusters, we observed that Cluster 0 was defined by high expression of CD19, CD20/MS4A1, CD72, CD79, and MHC class II, consistent with B cells, while Cluster 1 were comprised of cells with a transcriptome defined by the expression of NKG2A/KLRC1, CD94/KLRD1, NKG7, CD247, GNLY, and the cytotoxic molecule granzyme B (GZMB), consistent with an NK cell phenotype (**Fig 4C**).

To visualize the relationship of each cellular population, we color-coded cells according to their original sorting definition (i.e., B, NK, and NKB cells) and overlaid these on the sc-RNA-Seq feature plot (**Fig 4B**, bottom panel). As expected, the vast majority of cells (73 out of 78) that were sorted as CD20^+^NKG2A/C^-^ B cells were found in Cluster 0, conversely, 76 out of the 77 cells sorted as NKG2A/C^+^CD20^-^ NK cells were found in Cluster 1. However, as shown in **Fig 4B**, 80.5% (128 out of 159) of cells sorted using the CD20^+^NKG2A/C^+^ double-positive NKB definition had transcriptomes localized in the B cell-like Cluster 0, and relatively fewer cells (31 out of 159) in NK cell-like Cluster 1. The NKB cells from Cluster 0 showed higher expression of canonical B cell markers, while those from Cluster 1 showed higher expression of NK cell markers (**Fig 4D**). Notably, we also observed high expression of the prototypical cytotoxic molecules GZMB and perforin (PRF1) in cells from the NKB_1 cluster (**Fig 4D**).

We further investigated the transcriptome of NKB cells by examining our sorted population for co-expression of transcripts for canonical markers of NK cells (KLRC1) and B lymphocytes (MS4A1/CD20 or CD19) (**Fig 4E and 4F**). Of the cells sorted as NKG2A/C^+^CD20^+^ NKB cells, we detected MS4A1^+^KLRC1 co-expression (defined as MS4A1 > 2 reads/cell and KLRC1 > 2 reads/cell) in only 25 of 159 cells. Using the same cutoffs, 7 of 159 cells were detected to have co-expression of both CD19 and KLRC1. Lastly, we examined the fraction of NKG2A/C^+^CD20^+^ NKB cells for which co-expression of MS4A1/CD20, KLRC1, and IgH or IgL could be detected. As shown in **Fig 4G**, we detected co-expression of all three transcripts in only 8 of 25 NKG2A/C^+^CD20^+^ NKB cells. Overall, the transcriptomic data showed that the presence of a ‘true’ NKB cell phenotype was infrequent in spleen tissue of naïve RM.

### Splenic NKB cells contain functional immunoglobulin transcripts

**Fig 5:**
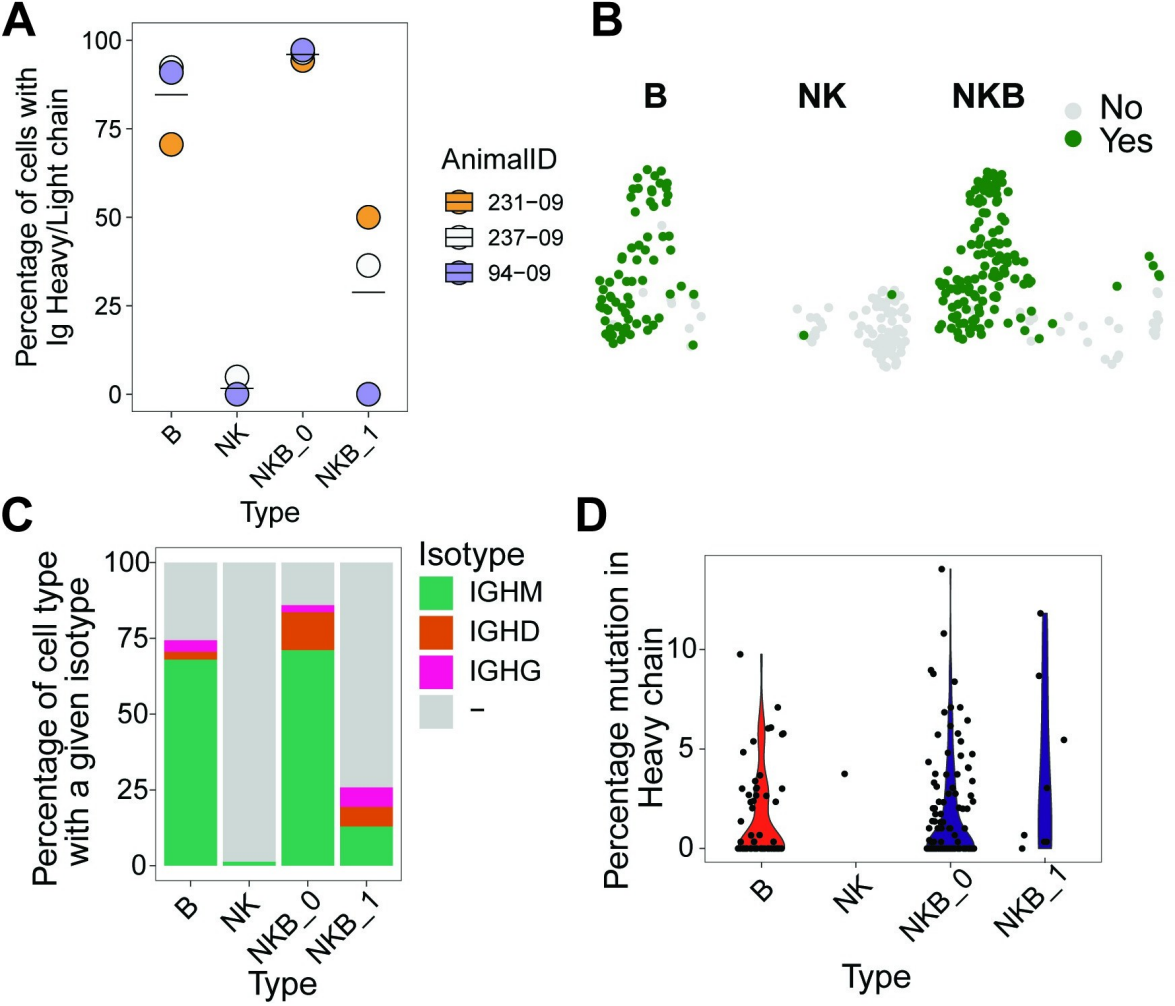
Expression of immunoglobulin transcripts in sorted NKB cells. Data from sc-RNA-Seq described in **Fig 4** were analyzed for immunoglobulin (Ig) transcripts representing functional antibody expression. **(A)** Percentage of cells in a given cell type where Ig heavy and/or light chain were recovered by BALDR pipeline in each rhesus macaque. **(B)** Expression of Ig chains. Colored cells in the UMAP represent ones from which a heavy and/or light chains was recovered by BALDR pipeline. **(C)** Isotype of Ig chains in each cell type. **(D)** SHM levels of Ig heavy chain in different cell types compared to KimDB v1.1 germline database.

A defining feature of B lymphocytes is the expression of rearranged Ig transcripts. To examine if NKB cells had detectable expression of antibody genes, we analyzed the sc-RNA-Seq data using the BALDR pipeline, which accurately reconstructs RM IgH and IgL chains from short-read sc-RNA-Seq data [21]. We quantified the frequency of cells with detectable IgH or IgL expression in each of the clusters (**Fig 5A and 5B**). We observed that an average 84.6% of cells sorted as CD20^+^NKG2A/C^-^ B cells had detectable expression of IgH, IgL or both chains; conversely, expression of either IgH or IgL transcripts in cells sorted as CD20^-^NKG2A/C^+^ NK cells was virtually undetectable, with only two out of 77 cells with detectable expression of Ig chains–one with only a light chain and the other with paired chains. When we examined the cells sorted as NKG2A/C^+^CD20^+^ NKB, we observed that the vast majority (123 of 128) of NKB cells with detectable Ig expression were localized to the B cell cluster/Cluster 0; in contrast, only 10 of 31 NKB cells localized to the NK cell cluster/Cluster 1 had detectable Ig expression.

In our previous work [1], we found that NKB cells in RM expressed IgM, IgG, and IgA isotypes on their surface. Therefore, we quantified the relative fraction of cells expressing each isotype in our data, and observed that 71% of NKB cells from Cluster 0 expressed IgM, followed by 12.5% expressing IgD and 2.3% IgG (**Fig 5C**). The isotype could not be determined for 14% of the NKB_0 cells. Similarly, for NKB_1 cells, the identified isotypes were IgM (4 cells), IgD (2 cells), and IgG (2 cells) with no determined isotype for the remaining 23 cells (**Fig 5C**). While we were unable to test the antigen-specificity of the reconstructed antibodies directly, we notably were able to find supporting evidence for most IgH transcripts in an independent database of RM immunoglobulin alleles (KimDB [22]).

Next, we tested if the Ig transcripts reconstructed by the BALDR analysis were representative of germline Ig alleles and assessed the degree of Ig somatic hypermutation (SHM) present in NKB cells using KimDB v1.1[22], a database of RM germline alleles (**Fig 5D**). We observed that for B cells, 53% cells had no mutations between the reconstructed sequence and germline IgH sequence, and the remaining had a mutation rate of 0.3–9.8%. In comparison, NKB cells in Cluster 0 had a similar distribution of mutations: 49% were perfect matches to the closest germline alleles, with mutations ranging from 0.3–14%. Additionally, the NKB cells in Cluster 0 had a similar frequency of cells with no mutations relative to B cells. Out of the eight cells from NKB cell Cluster 1, one was a perfect match to the closest germline allele and the mutations ranged from 0.3 to 11.8% in the others. Collectively, these data indicate that the IgH transcripts found within NKB cells contained V genes, which for the most part, were at or near 100% nucleotide identity with germline alleles. These data indicate that the rate of SHM was nearly indistinguishable between the NKG2A/C^+^CD20^+^ NKB cells and conventional B cells.

### NKB cells elevated early in mucosal SIV infection

**Fig 6:**
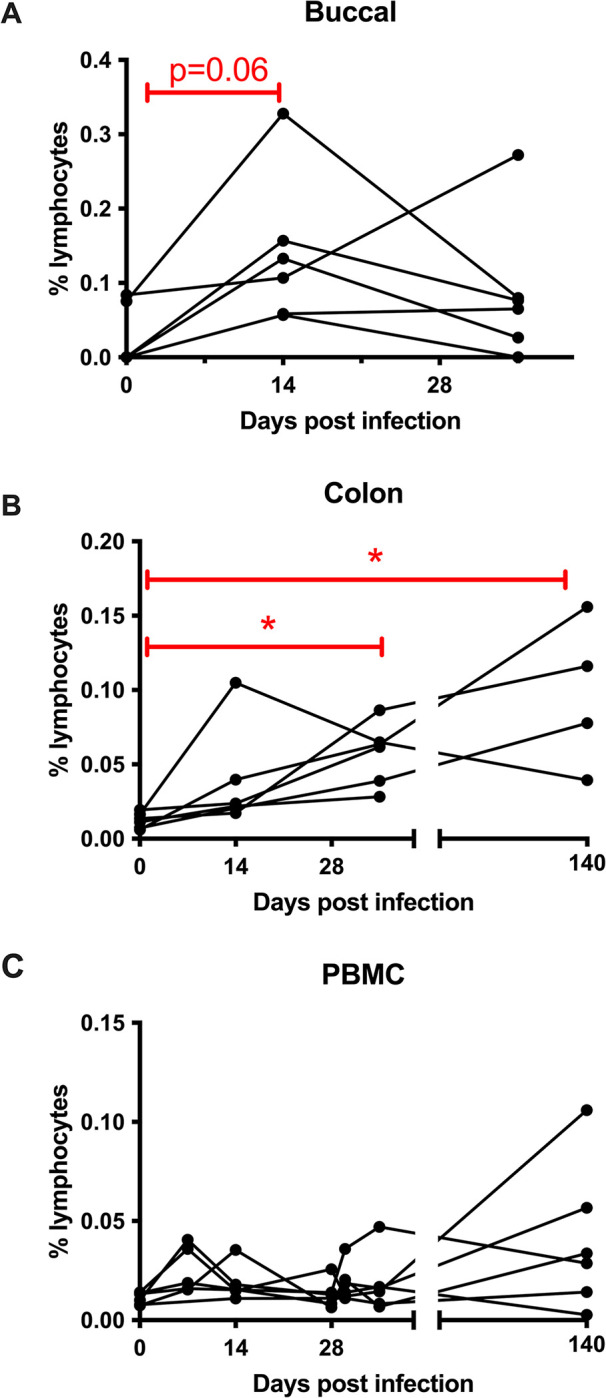
NKB cells in SIV-infected rhesus macaques (RM). Frequency of NKB cells in cells of **(A)** buccal tissue; **(B)** colon; and **(C)** peripheral blood mononuclear (PBMC) of RM at longitudinal time points post SIV infection. * indicates *p*-value < 0.05 by Mann-Whitney test.

Persistent mucosal inflammation during HIV infection, attributed to the immune response, significantly affects the gastrointestinal health of people living with HIV [23,24]. Recently, we and other researchers studied SIV-infected RM as an animal model of HIV, observing the presence of NKB cells within the chonically-infected gut that both produce inflammatory cytokines and proliferate during chronic infection, shedding light on their potential role [1,2]. Indeed, our previous research [1] has provided insight into the identification and systemic distribution of NKB cells in SIV-infected RM. Building upon our findings and drawing insights from other relevant studies [1,2,4] and to gain a comprehensive understanding of their involvement in SIV infection, we embarked on a longitudinal investigation of NKB cells at multiple timepoints following SIV infection in RM. Our findings revealed that NKB cells exhibited an expansion pattern, becoming notably prominent as early as day 14 in buccal tissue, although significance was not reached at this timepoint (*p* = 0.06) (**Fig 6A**). Their significant expansion was evident at day 35 and day 140 post-infection in the colonic mucosa of infected animals (**Fig 6B**), thereby indicating their potential roles in the dynamics of SIV infection. However, no significant differences in NKB cell frequencies were observed in PBMC (**Fig 6C**) and spleen [1], of SIV-infected RM.

### Colonic NKB cell frequencies associated with gut microenvironment in SIV infection

**Fig 7:**
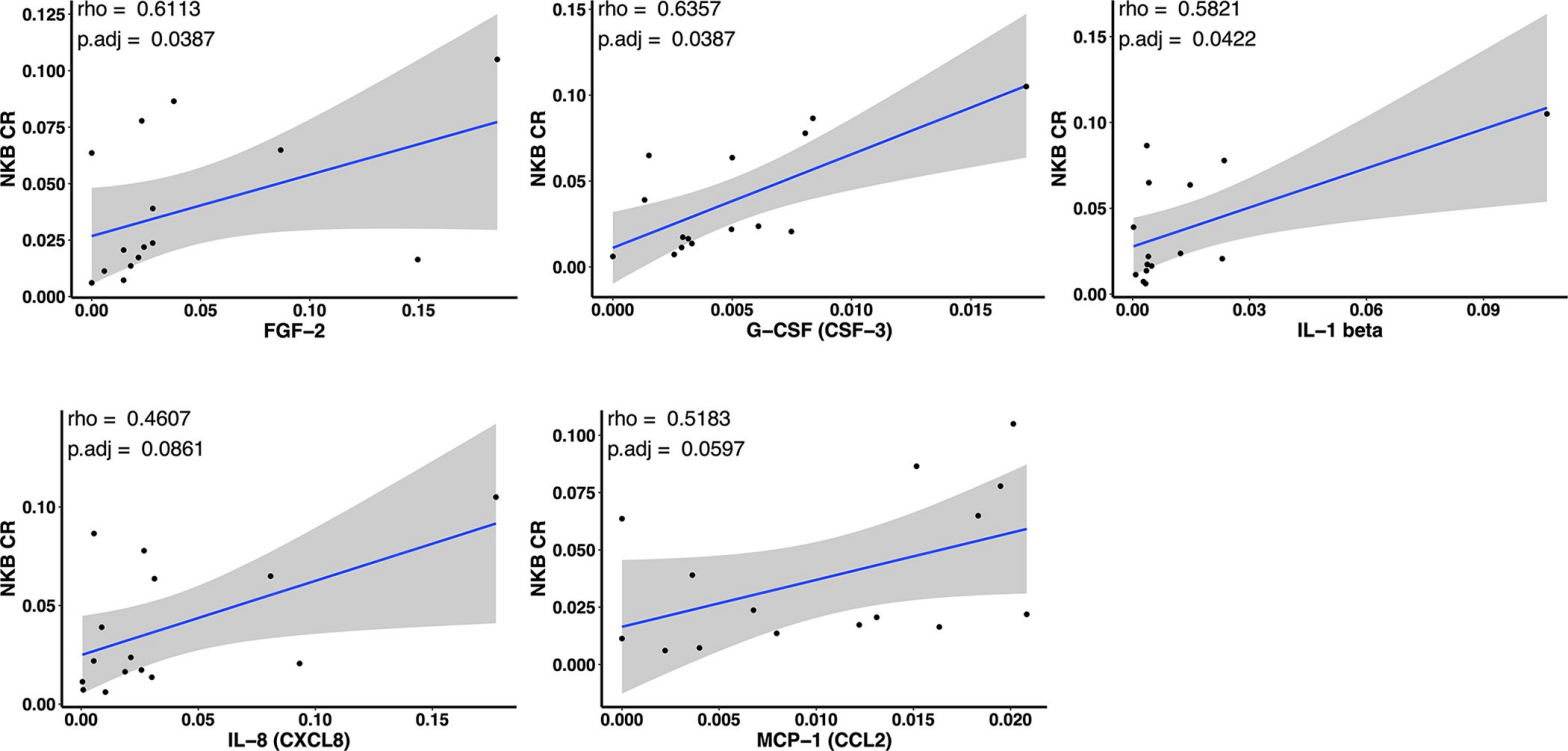
NKB cells associated with pro-inflammatory cytokines in SIV-infected colonic mucosa. Correlation graphs indicating associations of NKB cell frequencies in colorectal samples (NKB CR in x-axis) with respective cytokines including fibroblast growth factor (FGF)-2, granulocyte-colony stimulating factor (G-CSF), IL-1 beta, IL-8, and monocyte chemoattractant protein (MCP)-1 in pg/ml. Indicated rho and adjusted *p*-values were calculated by Spearman correlation test.

Being among the first responder cells during SIV infection, we hypothesized that NKB cells would be capable of modulating mucosal microenvironments through cytokine secretion in order to facilitate further innate and adapative immune responses against the pathogen. To test this, we quantified cytokines and chemokines in colorectal mucosal washes of SIV-infected animals using Luminex xMaP assay. Using Spearman correlation analysis, we identified significant positive associations between NKB frequencies from colorectal mucosa of SIV-infected animals and cytokines, including FGF-2 (rho = 0.611, adj p-value = 0.0387), G-CSF (rho = 0.6357, adj p-value = 0.0387), IL-1 beta (rho = 0.5821, adj p-value = 0.0422) (**Fig 7**). Similar positive associations of mucosal NKB cells were observed with IL-8 (rho = 0.4607, adj p-value = 0.0387) and MCP-1 (rho = 0.5183, adj p-value = 0.0597), cytokines which are responsible for cell migration, proliferation and inflammation [25,26], thus indicating recruitment of innate immune cells to the gut mucosa.

### SIV-mediated mucosal microbiome alterations correlate with peripheral NKB cells

**Fig 8:**
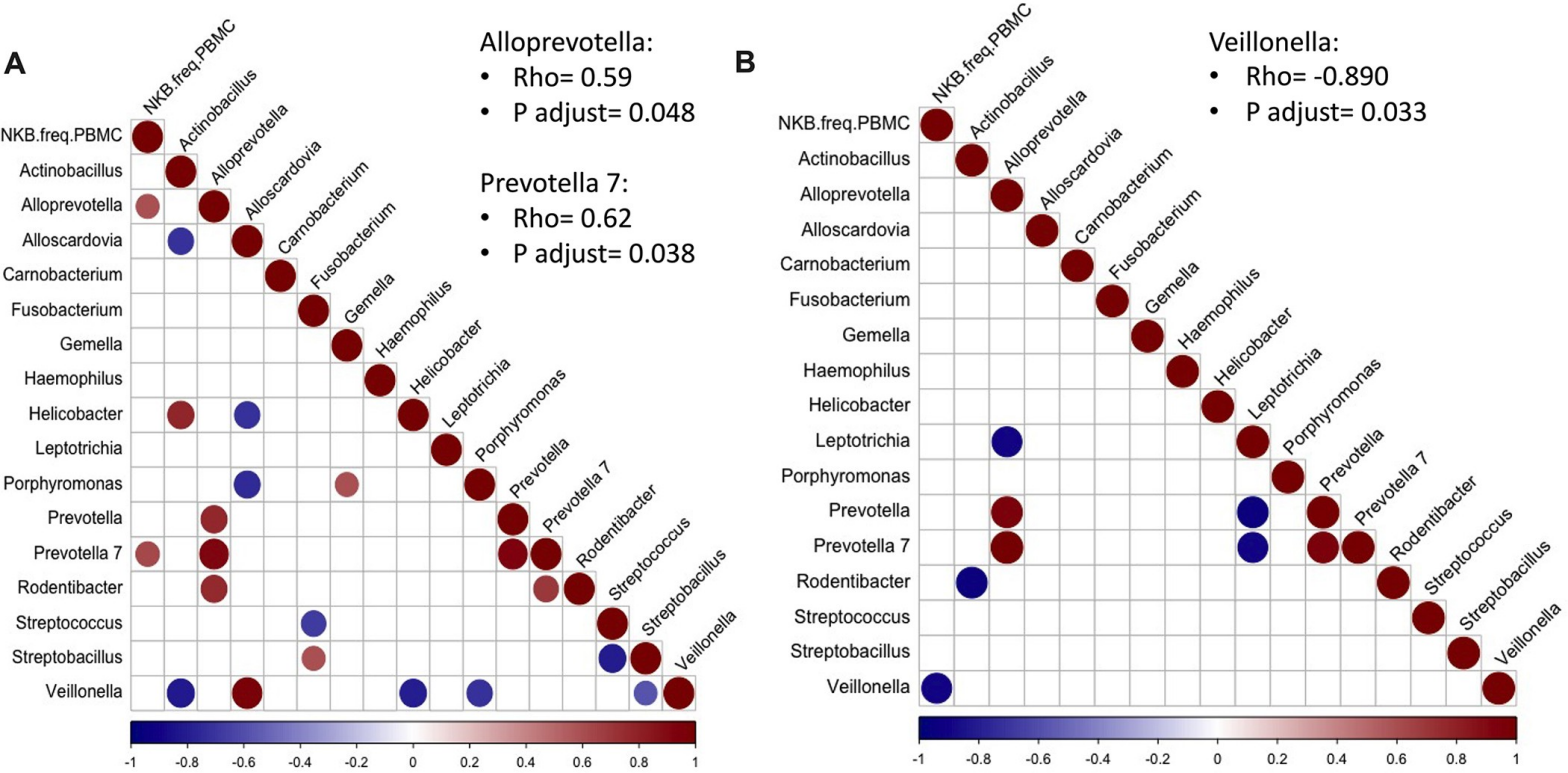
Oral microbiome associated with peripheral NKB cells in SIV infection. Relative abundance counts for top genus level bacteria in the buccal mucosa of SIV-infected rhesus macaques (RM) were selected for Spearman correlation analysis with NKB cell frequencies in the peripheral mononuclear blood cells of respective RM at (A) day 0 and day 14 and (B) day 14 post infection. Only correlations with adjusted *p*-value < 0.05 are shown in the graphs. The color scale indicates strength of correlation (rho values).

SIV/HIV infections have been known to alter both the gut and oral microbiomes [27–31]. In our previous study [32], we profiled bacterial communitites in buccal and colonic mucosa and identified Firmicutes and Epsilonbacteraeota phyla as the most abundant bacteria in oral and gut mucosa, respectively. To explore potential associations of microbiota modulations with NKB cells, we conducted a Spearman correlation to analyze the association of genus level bacterial taxa in the colonic and buccal mucosa with peripheral, buccal, and colonic NKB cells. Interestingly, we observed positive associations of peripheral NKB cells with *Prevotella 7* (rho = 0.62; adj p-value = 0.048) and *Alloprevotella* (rho = 0.59; adj p-value = 0.038) in the buccal mucosa of SIV-infected RM (**Fig 8A**), and negative association with *Velillonella* (rho = -0.89; adj p-value = 0.033) (**Fig 8B**) as early as two weeks post-infection, indicating imbalances in the oral microbiome associated with peripheral NKB cell frequencies during acute SIV infection.

## Discussion

Recently, NKB cells have gained recognized importance as first responder cells that initiate and activate the innate and adaptive immunity in inflammation and infections and have been proposed for therapeutic interventions [3]. NKB cells express both B and NK cell receptors, but they have been described to be distinct from both NK and B cell subsets. In mice, NKB cells expanded within 24 hours after microbial infection and mediated immune-activation of innate lymphoid cells and Th1 cells through their secretion of IL-18 and IL-12[9]. Data on NKB cells in humans and NHP is limited, however the the currently available studies have shown potential mucosal roles of NKB cells in colon of SIV-infected RM, pathogenic role in periodonitis in humans and potential roles in rheumatoid arthritis and liver infections/injury [1,2,4]. However, NKB cells, their phenotype, and their roles in homeostasis and disease are still not clearly understood due to multiple reasons, including the limited number of reports available. Another factor is the low frequency of NKB cells which are primarily enriched in tissues, specifically in spleen and lymphoid tissues. In order to fill in some of these gaps in understanding primate NKB cells, we analysed NKB cells from naïve and SIV-infected RM using flow cytometry, imaging cytometry, and transcriptomic aproaches.

Previous studies have shown that NKB cells have a phenotype that is distinct from NK and B cells due to their co-expression of both B cell-related markers (IgA, IgG, IgM, HLA-DR, and CD40) and NK cell receptors (CD56, CD16, and NKp46) [1,9]. In order to understand the trafficking and activation potential of NKB cells, we analyzed their expression of multiple surface proteins that are involved in trafficking and signaling in comparison to NK and B cells. Peripheral NKB cells at homeostasis expressed high Syk and low Zap70, CD3z, and FcRg which were similar to B cell signaling proteome expression. Similarly, NKB trafficking markers, which were high in CD62L and low in CD69 and a4b7, were similar to B cells and converse to NK cell trafficking phenotypes. These data further confirm that the NKB cells are most likely a subset of innate like B cells, as described by multiple groups [11,13,14,33]. Since NKB cells in naïve PBMC are low in frequency, and NKB have been studied primarily in tissues, we used imaging techniques for their *in situ* localization in lymphoid and mucosal tissues at homeostasis. While NKB cells were enriched in spleen and MLN, we identified lower frequencies in jejunum and colon and even fewer cells in liver of naïve RM, thus corroborating with other reports indicating the presence of unique innate cells based on surface protein expression patterns. Interestingly, upon SIV infection, we observed expansion of NKB cells in both buccal and colonic mucosa of RM, thus indicating a potential roles for this subset in mucosal immunity.

We next set out to examine NKB single-cell transcriptomic signatures to confirm similarities to either B or NK cells, or if they possess their own distinct signature. The vast majority of sorted NKB cells were clustered with B cells as opposed to NK cells. Upon examination of typical NK cell markers such as KLRC1, NKG7, and KLRD1, the majority of NKB cells did not express significant levels of these NK cell-specific genes above background. However, when examining B cell markers such as CD19, MS4A1/CD20, and CD79A, we observed that NKB cells express high levels of these genes. These findings further suggest that NKB cells are more likely to be a subset of B cells that have gained expression of certain NK cell receptors. Further strengthening this conclusion is the fact that NKB cells had detectable levels of functional heavy and light Ig chains. The transcription factor EOMES, which is critical for NK cell development, was not observed to have appreciable transcript levels in the majority of NKB cells, further suggesting that these cells do not orginate from an NK cell lineage. However, we did observe that minority of cells with the NKB cell phenotype (approximately 20%) clustered with and expressed transcripts more similar to NK cells. It is currently unclear whether these cells originated with the other NKB cell subset. It is possible that these two subsets originated from a common progenitor and represent different lineages, or that they could represent different maturation stages of the same lineage. Additionally, it is possible that these subsets are independent and convergently developed to express both B and NK cell receptors. Therefore, more studies are needed to clearly identify and characterize the true NKB cells.

Our study shows that the NKB immune cell subset, while unique, is more similar to B cell subsets, and is enriched in spleen and MLN during homeostasis. Transcriptomic data showed that “bona fide” NKB cells, i.e., cells with appreciable transcript levels of both B and NK cell hallmark genes, comprised only 20% of the total population of cells expressing the NKB surface phenotype (lin-CD20^+^NKGA/C^+^), thus undermining the functional relevance of this subset during infections and potential translation to therapeutic/vaccine development. While our data showed that NKB cells were at low frequencies, we did observe expansion of NKB cells in the colonic mucosa of SIV-infected RM, indicating potential modulation and functions of the NKB cell population in infected tissues. However, this leads us to question what percentage of this expanded population are of the true NKB cell phenotype, and what functions can be directly attributed to them. Functionally, NKB cells have been described as expressing several cytokines, including IL-1b, IL-6, IL-12, and IL-15, upon stimulation/infection, in addition to their signature IL-18 secretion [9]. Indeed, colonic NKB cells in SIV-infected RM were positively correlated with pro-inflammatory cytokines (IL-1b, IL-8, and MCP-1) and mediators of cell proliferation and recruitment (FGF-2 and G-CSF) in colorectal washes. IL-1b is a critical inflammatory cytokine for protection against viruses and pathogens. IL-8, G-CSF, and MCP-1 are chemoattractants for myeloid cells, including neutrophils and monocyte/macrophages [34–36], which enable infiltration of innate immune effectors to the colonic mucosa. FGF-2, secreted by endothelial cells and fibroblasts involved in recruitment of immune cells to inflamed sites for wound healing [37], has been reported in the circulation of people living with HIV (PLWH) and is associated with kidney pathologies [38]. Considering these factors, we postulate that elevated NKB cells in mucosal infections could modulate the mucosal microenvironment towards an inflammatory mileu unfavorable for pathogens, although uncontrolled immune cell recruitment and functions could cause tissue damage.

Altered mucosal microbiota and microbial translocation are hallmarks of HIV infection [27,39]. PLWH are highly susceptible to oral infections and altered oral microbial dysbiosis, leading to gingivitis and periodontitis, which in turn can cause loss of periodontal ligament, alveolar bone structure, and loss of teeth [40–43]. Indeed, NKB cells have been implicated in the immunopathogenesis of periodontitis. In a murine model of chronic periodontitis induced by *P*. *gingivalis* and in acute periodontitis patients, NKB cells were elevated and induced inflammation through secretion of IL-18 and the neutralization of IL-18 prevented bone resorption and inflammation [4]. In SIV-infected RM, we observed positive correlation of peripheral NKB cells with the genera *Alloprevotella* and *Prevotella*, both of which play important roles in carbohydrate metabolism and fermentation processes and have been implicated in oral microbial dysbiosis and periodontitis [44,45]. Interestingly, *Veillonella*, which has been reported to increase with poor levels of oral hygiene [46] and promotes intestinal inflammation [47], was negatively associated with peripheral NKB cell frequencies in SIV-infected RM, indicating that the differential microbial dysbiosis and immunopathogenesis of oral microbiome could be mediated by NKB cells during SIV infection.

Through multiple techniques, which included imaging, flow cytometry, and transcriptomic analysis, we confirmed NKB cells as an unique subset of innate immune cells that exist in homeostasis. The incidence of NKB cells expanded within SIV-infected mucosae and were associated with an altered mucosal microbiome of dysbiosis and cytokine milieu, boosting immune cell recruitment, indicating that NKB cells play a role in oral dysbiosis and pathogenesis within the gut during SIV infection. However, there is still much to learn about NKB cells, given their rarity in homoestasis and infection. To better understand these unique and innate NKB cells, we must first develop a strategy to truly define and potentially utilize the NKB cell phenotype that matches their transcriptomic profile, since definitions of NKB cells using surface phenotyping alone likely overestimate the frequency of this population. Future studies aimed at understanding the pathophysiological role of this subset in infections and inflammatory conditions will likely require transcriptomic definitions and additional functional characterization.

## Methods

### Ethics statement

Indian-origin rhesus macaques (*Macaca mulatta*, RM) were housed at a Biomere facility (Worcester, MA), and all studies were carried out in strict accordance with the ethical principles outlined in the U.S. National Institutes of Health Guide for the Care and Use of Laboratory Animals with recommendations of the Weatherall report; “The use of non-human primates in research”[48]. All blood and biopsy samplings collected as part of study protocol #16–08. Protocol #16–08 was reviewed and approved by the Biomere Institutional Animal Care and Use Committee.

### Animal welfare and study

The diet of the RM included standard monkey chow diet supplemented daily with fruit and vegetables and water ad libitum. Social enrichment was provided to the animals and was overseen by veterinary staff. Animal health was monitored daily and if any signs of significant weight loss, disease or distress, they were evaluated clinically and then provided dietary supplementation, analgesics and/or therapeutics as necessary. RM were chronically infected with SIVmac251 intrarectally (n = 6) until 6 months post infection. Blood, biopsies, and tissues were collected longitudinally and processed using standard protocols to obtain mononuclear cells which were frozen for later use [1]. Similarly, samples were obtained from naïve RM and stored for normal baseline control.

### Flow cytometric staining and analysis

Frozen PBMC and mononuclear cells from buccal and colonic tissues of naïve and SIV infected RM were thawed and stained with aqua dye for 20 minutes at room temperature for live/dead cell discrimination followed by antibody master mix containing surface markers which was then incubated for 20 minutes at room temperature. Following this, cells were stained intracellularly for signaling proteins at 4°C for 20 minutes. Markers used for staining are listed in **S1 Table**. Samples were then washed twice with wash buffer and fixed with 1% formaldehyde. Fixed samples were recorded using BD Symphony flow cytometer (BD Biosciences) and analyzed using FlowJo software (version 10.6.1). T- Stochastic Neighbor Embedding (tSNE) analysis was performed on the flow cytometric data using Barnes-Hut-Stochastic Neighbor Embedding (bh-SNE) approximations.

### ImageStream staining and analysis

Mononuclear cells from spleen of SIV-naïve RM were stained with primary antibody staining which included: CD3 (clone SP34.2; manufacturer BD Pharmingen), CD14 (M5E2; BD Pharmingen), CD20 (2H7; BioLegend), HLA-DR (G46-6; BD Pharmingen), and NKG2A (Z199; Beckman Coulter). After incubation for 20 minutes at room temperature, the cells were washed with wash buffer (1XPBS containing 2% FBS) and stained with DAPI dye (ThermoScientific) for live and dead cell discrimination. After 5 minutes of incubation, cells were washed and fixed with 1% formaldehyde. Samples were recorded using an ImageStreamX Mk II (EMD Millipore) and analyzed using IDEAS Application v6. The general analysis, colocalization and internalization modules in the IDEAS software were utilized in analyzing these samples.

### ChipCytometry

Tissue sections from frozen spleen, MLN, and jejunum of naïve animals were cut onto coverslips (Canopy Biosciences) and after overnight incubation at -80°C, the sections were fixed with acetone and washed with 90% alcohol, 70% alcohol and ZELLKRAFTWERK wash buffer on ice. Sections were assembled into tissue chips (ZellSafe Tissue Chips) and filled with storage buffer. The chips were then washed and subjected to iterative multiplex staining with antibodies (**S2 Table),** imaging and photobleaching using Zellscanner microscope (Canopy Biosciences). The images were analyzed using the ZKWApp and ImageJ software [49]. The images were converted to FCS files and analyzed using ZKWApp.

### Cell sorting for RNA-seq

We sorted CD45^+^CD3^-^NKG2A/C^+^CD20^+^ NKB cells from three SIV-negative RM as single cells into individual wells of 96-well plates using a BDFACS AriaII flow cytometer. For comparison, we also sorted peripheral blood total B lymphocytes (which was comprised of both memory and naïve B cells, CD45^+^CD3^-^NKG2A/C^-^CD20^+^), and NK cells (CD45^+^CD3^-^NKG2A/C^+^CD20). For this comparison, we utilized a sc-RNA-Seq method in which single cells were sorted into individual wells and sequencing libraries were prepared and sequences using the Smart-Seq method by Medgenome.

### Single-cell RNA-Seq analysis

The BALDR pipeline was used to reconstruct Ig chains from all samples using the FilterNonIG method [21]. For some samples, the depth of sequencing was very large and these were downsampled to 2 million reads. The RM reference from Cirelli *et al*.[50] and Ramesh *et al*.[51] were used for VDJ and constant region genes respectively as part of the BALDR pipeline. The fastq files were aligned to MacaM v7[52] using STAR aligner v 2.5.2b[53]. The raw counts from ReadsPerGene output of STAR was used for downstream analysis using Seurat v4.0.4[54]. Two plates were dropped due to issues with quality. The cells were filtered using the following criteria: nFeature_RNA > 500, nFeature_RNA < 7500 & nCount_RNA < 1.5x10^6^ giving a total of 314 cells (B cells– 78, NK cells– 77 and NKB cells—159). The cells were normalized using the NormalizeData function with a scaling factor of 1x10^6^. The vst method was used to find variable features and the ScaleData function was used to regress potential batch effects between plates. The RunUMAP function was used for dimensionality reduction using the first 45 dimensions and seurat clustering was performed using a resolution of 0.3. The FindMarkers function was used to obtain differentially expressed gene between clusters 0 and 1 using the MAST method [55]. The mutations were determined using igblast version 1.21.0[56] against the RM Ig germline database KimDB v1.1[22].

### Luminex xMAP assay for colorectal washes

Cryopreserved colorectal wash samples were thawed at room temperature and centrifuged at 1500g for 5 minutes to remove any fecal sediments. The clear supernatants were then used to assess the concentrations of cytokines and chemokines utilizing the Procartaplex NHP Cytokine/Chemokine 37-plex kit (ThermoFisher, Catalog number EPX370-140045-901) in accordance with manufacturer’s instructions. Briefly, vortexed magnetic capture beads (50μL) were added to sample wells in a 96-well optical plate and washed using wash buffere provided in kit and a handheld magnet. Samples were added as duplicates at 50 μL volume/well to the assigned wells. Duplicate wells containing 50 μL of phosphate-buffered saline (PBS) were used as background measurements. For generation of standard curves, manufacturer-provided lyophilized standards were reconstituted in PBS and four-fold serial dilutions for a total of nine standards were added in duplicates as per manufacturer protocol.

The plate was then sealed, placed on a shaker at 500rpm for 20 hours at 4°C. On the next day, the plate was washed twice with wash buffer and 25μL of detection antibody cocktail was added to all wells and placed on a shaker (500rpm) for 30 minutes at room temperature. Following incubation, the plate was washed twice and incubated with 50μL of Streptavidin-PE on the shaker (500rpm) for 30 minutes. Finally, the plate was washed twice, and 120μL of reading buffer was added and incubated on shaker (500rpm) at room temperature for 5 minutes before being analyzed on the Luminex200 instrument. Measurements were calculated and reported with the xPONENT 4.2 software (Luminex Corporation).

### Mucosal microbiome analysis

Snap-frozen colon and buccal biopsies were subjected to DNA extraction and 16S RNA sequencing and analyzed as previously described [32] (NCBI SRA gene bank bioproject number: PRJNA688737). Spearman correlations were performed to explore associations between the top relative abundant bacteria and NKB cell frequencies at specific time points. Microbiome data used for analysis were previously processed and analyzed in published research exploring the effect of a oral probiotic supplementation on early SIV infection of rhesus macaques [32]. Correlation statistics were performed in R utilizing the rstatix package. P-values underwent adjustment using the Benjamini-Hochberg method with an alpha set at 0.05[57]. Correlation plots were generated with the ggplot2 package in R [58].

### Statistical analysis

Statistical signficance of difference in expression of trafficking and signaling markers of NKB cells in comparison to B and NK cells in PBMC were determined by two-way ANOVA. Median rank differences between cells/mm2 and percentages of NKB, B, and NK cells in multiple tissues of naïve animals, and median rank differences of NKB cell frequencies in buccal and colon tissues as well as PBMC at different time points of SIV infection in comparison with baseline at day 0 were analyzed by non-parametric Mann-Whitney test. Statistical analysis were performed using GraphPad Prism software. Differences were considered significant when the *p*-value was < 0.05.

For Luminex data, NKB cell frequencies and Luminex cytokine concentrations (pg/mL) from colorectal tissue samples were loaded into R [59]. Cytokine levels were ordered by variance across the entire dataset and the top five highest variance cytokines were chosen for Spearman correlation. Spearman rho- and p-values were generated with the cor.test method in the base stats [59] package using the ‘Spearman’ method. P-values were then adjusted using the p.adjust method in the base stats [59] package using the Benjamini-Hochberg [57] method. Plots were generated with the ggplot2 package [58].

## Supporting information

S1 Fig Gating strategy for NKB cell sorting. Spleen mononuclear cells from naïve rhesus macaques were stained for flow cytometric sorting. From the Live CD45^+^CD3^-^CD14^-^ subset, NKB cells were sorted as NKG2A/C^+^ CD20^+^ cells, NK cells as NKG2A/C^+^CD20^-^, and B cells as NKG2A/C^-^CD20^+^ cells.
(TIF)

S2 Fig Images from ChipCytometric analysis of mesenteric lymph nodes (MLN). Images show individual marker expression obtained by cycles of staining, imaging, and bleaching at a single position of MLN tissue of a naïve rhesus macaque obtained via ChipCytometry.
(TIF)

S1 Table List of antibodies used. (DOCX)

S2 Table Staining plan for ChipCytometry. (DOCX)

S1 Data Trafficking and signaling markers. (XLSX)

S2 Data NKB frequencies by ChipCytometric imaging. (XLSX)

S3 Data NKB frequencies by flow cytometry. (XLSX)

S4 Data Cytokine MFI by Luminex. (XLSX)

S5 Data RNAseq IGH filtered data. (XLSX)

S6 Data RNAseq IGKL filtered data. (XLSX)
