# Supplementary figures and images for: Natural killer-like B cells are a distinct but infrequent innate immune cell subset modulated by SIV infection of rhesus macaques
Source: PLoS Pathog. 2024 May 13;20(5):e1012223. doi: 10.1371/journal.ppat.1012223 (PMC11115201; doi:10.1371/journal.ppat.1012223)

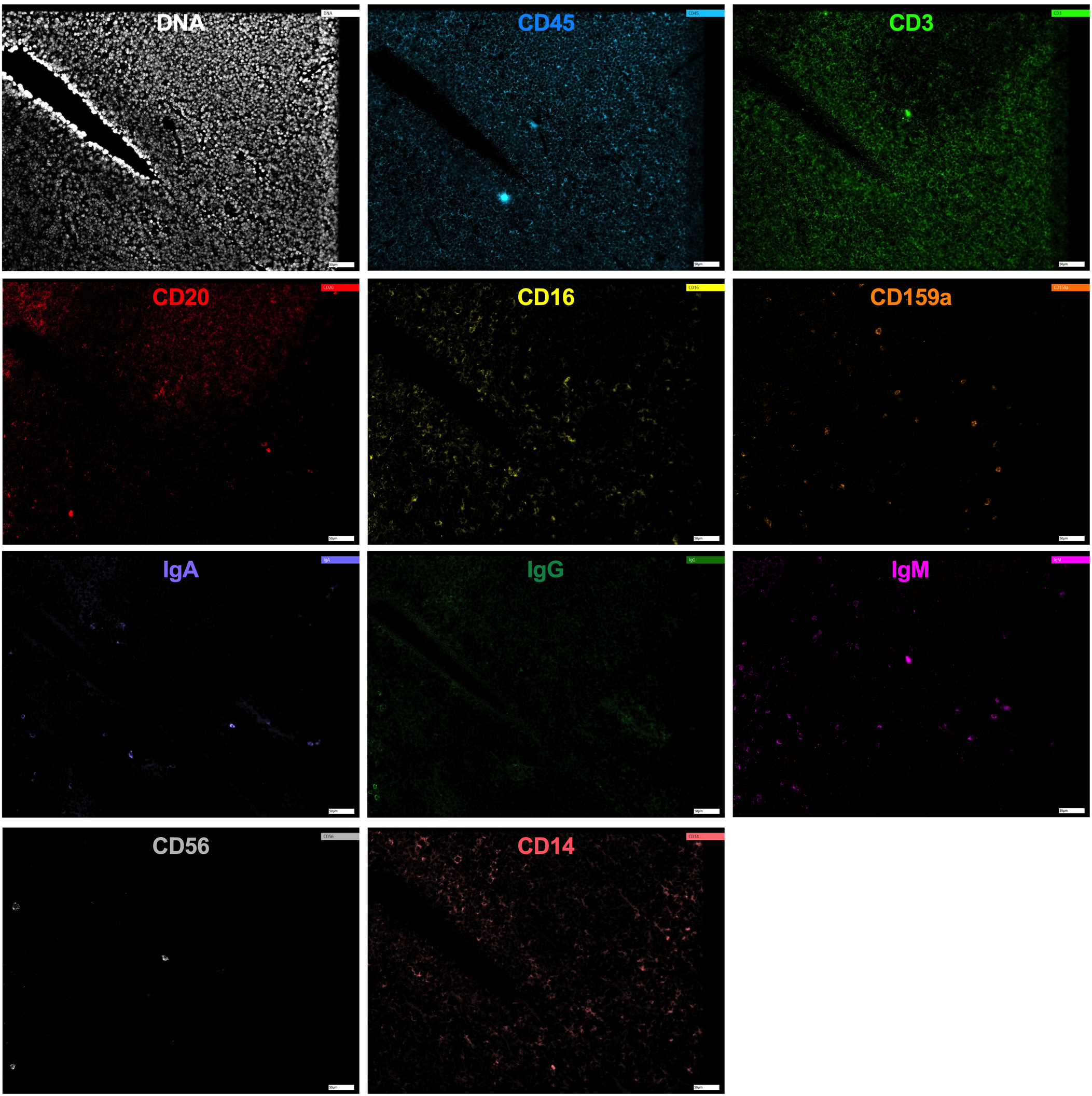

Supplement: S1 Fig — Spleen mononuclear cells from naïve rhesus macaques were stained for flow cytometric sorting. From the Live CD45+CD3-CD14- subset, NKB cells were sorted as NKG2A/C+ CD20+ cells, NK cells as NKG2A/C+CD20-, and B cells as NKG2A/C-CD20+ cells. (TIF) [file ppat.1012223.s001.tif]

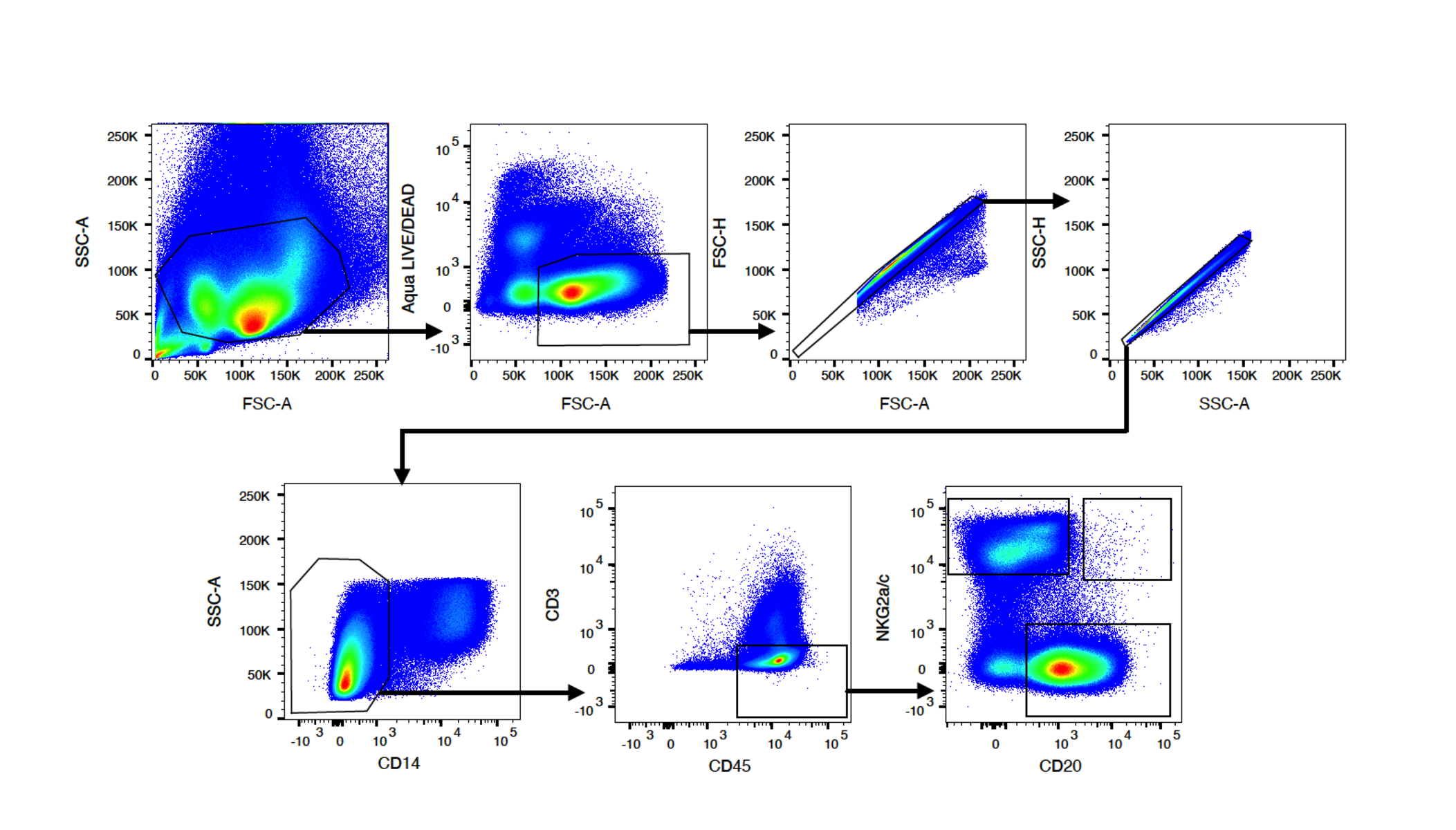

Supplement: S2 Fig — Images show individual marker expression obtained by cycles of staining, imaging, and bleaching at a single position of MLN tissue of a naïve rhesus macaque obtained via ChipCytometry. (TIF) [file ppat.1012223.s002.tif]
