# Supplementary material for: Natural killer-like B cells are a distinct but infrequent innate immune cell subset modulated by SIV infection of rhesus macaques
Source: PLoS Pathog. 2024 May 13;20(5):e1012223. doi: 10.1371/journal.ppat.1012223 (PMC11115201; doi:10.1371/journal.ppat.1012223)
Supplement: S1 Table — (DOCX) [file ppat.1012223.s003.docx]

**Supplementary Table 1 : List of antibodies used**

| **ANTIBODIES (CLONE)** | **SOURCE** | **IDENTIFIER** |
| --- | --- | --- |
| α4β7-APC (A4B7) | <http://nhpreagents.org/NHP/default.aspx> | Cat# PR-1421 |
| CD3-BV786 (SP34.2) | 3D Biosciences | Cat# 563918; RRID: AB_2738487 |
| CD14-BUV395 (MϕP9) | BD Biosciences | Cat# 563561; RRID: AB_2744288 |
| CD16-BUV496 (3G8) | BD Biosciences | Cat# 564653; RRID: AB_2744294 |
| CD20-BUV395 (L27) | BD Biosciences | Cat# 740204; RRID: AB_2739954 |
| CD56-BV786 (NCAM16.2) | BD Biosciences | Cat# 564058; RRID: AB_2738569 |
| CD62L-BV711 (SK11) | BD Biosciences | Cat# 565040; RRID: AB_2744438 |
| CD69-ECD (TP1.55.3) | Beckman Coulter | Cat# 6607110; RRID: AB_1575978 |
| CD159a/NKG2a-APC (Z199) | Beckman Coulter | Cat# A60797; RRID: AB_10643105 |
| CD159a/NKG2a-PC7 (Z199) | Beckman Coulter | [Cat# B10246; RRID: AB_2687887](https://www.ncbi.nlm.nih.gov/nuccore/B10246) |
| CD247/CD3ζ-FITC (H146-968) | ThermoFisher Scientific | Cat# MA5-17673; RRID: AB_2539063 |
| CD335/NKp46-PC5 (BAB281) | Beckman Coulter | Cat# A66904 |
| CD335/NKp46-PC7 (BAB281) | Beckman Coulter | [Cat# B38703](https://www.ncbi.nlm.nih.gov/nuccore/B38703) |
| CD335/NKp46-PE (BAB281) | Beckman Coulter | Cat# IM3711; RRID: AB_1575960 |
| FcR (γ-chain)-FITC (rabbit polyclonal) | Millipore Milli-Mark | Cat# FCABS400F; RRID: AB_11203492 |
| FcR (γ-chain)-Alexa Fluor700 (rabbit polyclonal) | Millipore Milli-Mark | Conjugated in-house |
| HLA-DR-ECD (HLA-DR) | Beckman Coulter | Cat# IM3636; RRID: AB_10643231 |
| Syk-PE (4D10.1) | ThermoFisher Scientific | Cat# 17-6696-42; RRID: AB_10714836 |
| ZAP70-Pacific Blue (1E7.2) | ThermoFisher Scientific | Cat# MHZAP7004-4; RRID: AB_2539777 |
