# Supplementary material for: Natural killer-like B cells are a distinct but infrequent innate immune cell subset modulated by SIV infection of rhesus macaques
Source: PLoS Pathog. 2024 May 13;20(5):e1012223. doi: 10.1371/journal.ppat.1012223 (PMC11115201; doi:10.1371/journal.ppat.1012223)
Supplement: S2 Table — (DOCX) [file ppat.1012223.s004.docx]

**Supplementary Table 2:** Staining plan for ChipCytometry

| **Cycle** | **Epitope** | **Clone** | **Conjugate** | **Dilution** | **Protocol** |
| --- | --- | --- | --- | --- | --- |
| Cycle 1 | CD20 | LT20 | PE | 1:100 | 15 Min Surface |
|  | CD3 | SP34.2 | BUV395 | 1:1000 |  |
|  | IgM | 2C11-1-5 | FITC | 1:1000 |  |
|  | CD16 | 3G8 | PerCP/Cy5.5 | 1:60 |  |
| Cycle 2 | IgA | IgA5-3B | Biotin | 1:100 | 15 Min Surface x2 |
|  | Anti-Biotin | 1D4-C5 | PE | 1:1000 |  |
| Cycle 3 | NKG2A (CD159a) | REA110 | PE | 1:200 | 15 Min Surface |
|  | CD56 | HCD56 | PerCP/Cy5.5 | 1:200 |  |
|  | CD45 | D058-1283 | BUV395 | 1:600 |  |
| Cycle 4 | CD14 | M5E2 | PerCP/Cy5.5 | 1:100 | 15 Min Surface |
|  | IgG | Polyclonal | PE | 1:10000 |  |
| Cycle 5 | Hoechst | DNA | BUV395 | 1:500,000 | 5 min RT |
